# Prediction of quality attributes (mechanical strength, disintegration behavior and drug release) of tablets on the basis of characteristics of granules prepared by high shear wet granulation

**DOI:** 10.1371/journal.pone.0261051

**Published:** 2021-12-09

**Authors:** Amjad Khan

**Affiliations:** Department of Pharmacy, Kohat University of Science and Technology (KUST), Kohat, Pakistan; St. John’s University College of Pharmacy and Health Sciences, UNITED STATES

## Abstract

High shear wet granulation is commonly applied technique for commercial manufacturing of tablets. Granulation process for tablets manufacturing is generally optimized by hit and trial which involves preparation of granules under different processing parameters, compression of granules and evaluation of the resultant tablets; and adjustment is made in granulation process on the basis of characteristics of tablets. Objective of the study was to optimize the process of high shear wet granulation and prediction of characteristics of tablets on the basis of properties of granules. Atenolol granules were prepared by high shear wet granulation method, using aqueous solution of polyvinyl pyrrolidone (PVP k-30) as binder. Concentration of binder solution and granulation time were taken as process variables, both studied at three levels. Different combinations of process variables were determined by Design Expert software. Granules were evaluated for different parameters on the basis of SeDeM-ODT (Sediment Delivery Model-Oro Dispersible Tablets) expert system. Granules from all the trials were compressed using round (10.5 mm) flat faced punches at compression weight of 250 mg/tablet. Tablets were evaluated of different quality control parameters as per USP. Results showed that both the process variables had positive effect on mechanical strength of tablets and negative effect on disintegration and dissolution rate. Granule prepared with highest level of binder concentration (15%) and highest granulation time (60 sec) resulted in tablets with highest crushing strength (11.8 kg), specific crushing strength (0.328 kg/mm^2^), tensile strength (0.208 kg/mm^2^), lowest value of friability (0.19%) and highest disintegration time (10.9 min), as predicted from granules characteristics on the basis of SeDeM-ODT expert system. Drug release from Trial-13 (processed under highest level of both process parameters) was also lower than rest of the trials. It is concluded from the study that quality characteristics of tablets can be predicted from granules characteristics using SeDeM-ODT expert system. Furthermore, SeDeM-ODT expert system can also be used for optimization of the process of high shear wet granulation.

## Introduction

In the science of pharmaceutics, a drug is transformed into an appropriate delivery system which is convenient and safe for the patient, and suitable for large scale manufacture [1]. Tablet is one of the highly used dosage form and is prepared by compression of powdered ingredients by a compression machine. As a real physical system, particles are in contact with the neighboring particles and subjected to various surface forces which gives tablets an integrated shape [2]. Tablets are expected to maintain their mechanical integrity, without compromising their disintegration behavior and drug release. Quality attributes of tablets depends upon;

- Characteristics of API
- Characteristics of excipients
- Mixing of different ingredients (API and excipients)
- Process of granulation
- Processing parameters during compression

Most of the powdered materials lack sufficient compressibility and/or flowability, and are granulated. During granulation, particles of powder are brought to-gather, and bond are created, with the help of a binding agent [3–6]. Three types of bonding mechanisms have been reported for formation of pharmaceutical granules, as;

- **Distance forces**: Distance forces are affected by distance between the two interacting surfaces and active at a distance of 1 x 10^3^ Å. These include Vander Waal’s forces and electrostatic force of attraction [7].
- **Solid bridge**: Solid Bridge is the area between two particles where particles are in continuous phase by their partial fusion [8]. It is the strongest form of bond in pharmaceutical granules or compacts
- **Mechanical interlocking**: Mechanical interlocking is observed among fibrous or irregularly shaped ingredients, when two particles are hooked and twisted to gather in closely packed system [7].

During granulation and subsequent compression, these different mechanism of attraction are involved simultaneously, depending upon nature of ingredients, preparation process and processing parameters [8,9]. Mechanical characteristics are of prime importance to determine in order to identify the properties critical for the tableting behavior in terms of compressibility and compact-ability. Hence, testing of physical and mechanical properties of drugs and excipients represents an import activity during the formulation of tablets [10]. Tablet structure and strength during compression can be affected by modifying structural and mechanical properties of particles. It has been reported that variations in such properties will affect the degree of deformation under compression force and hence the structure of the formed tablets [11]. Along with API, tablets contain excipients, required for specific functionality during tablet preparation like disintegration, lubrication and binding action. Usually poor characteristics of API are masked with excipients. But in case of large dose of API with poor flow and compressibility, wet granulation is the only solution [12]. During formulation development by wet granulation technique, the most critical step is optimization of granules characteristics which govern all the quality parameters of tablets. In near past the granulation process has been improved and a number of equipments are available for wet granulation which has reduced the process time, number of steps, material handling, and energy consumption [13]. As the process of wet granulation has been made speedy so the process parameters and formulation factors need to be critically controlled. The process of high shear wet granulation is normally optimized by hit and trial, which involves preparation of granules under different processing parameters, granules are compressed, the resultant tablets are evaluated and adjustment is made in granulation process on the basis of characteristics of tablets. It makes formulation development a tedious, requires enormous efforts and time consuming job. There was need for an optimization process which can predict quality characteristics of tablets at granules level, without any compression. Objective of the study was to predict characteristics of the tablets on the basis of the granules properties, which will help in optimization of granulation process and will lead to enhanced robustness of tablet preparation. Atenolol was selected as model drug on the basis of its large dose (100 mg/tablet), poor flow and compressibility. It is an off-white crystalline powder [14]. Atenolol is a β-adrenergic blocker, available in tablet dosage form. Chemically, atenolol is 2-(4-{2-hydroxy-3-[(propan-2-yl) amino] propoxy} phenyl) acetamide, having a molecular weight of 266.311 g/mol. It is a BCS class-III drug with good water solubility and poor permeability [15]. In the present study, granules were prepared by standard wet granulation technique, under different processing parameters (binder concentration and granulation time). Granules were evaluated by SeDeM-ODT expert system and characteristics of the resultant tablets were predicted. Granules were compressed and tablets were evaluated for various quality control parameters.

## Material and methods

### Material

Atenolol (Wuhan Biocare Bio-Pharm Co., Ltd., China) was purchased from Nenza Pharmaceuticals Pvt. Ltd. Peshawar, Pakistan. Micro crystalline cellulose (F.M.C. International, Ireland), lactose (Molkerei Meggle, Germany), magnesium stearate (Coin Powder International Company Ltd, Taiwan), and primojel (sodium starch glycolate; F.M.C International, Ire Land) were purchased from local market of Peshawar, Pakistan. Purified water was prepared by Milli-Q^®^ system (Millipore, Milford, MA, USA).

### Preparation of granules

The process of granulation by high shear wet granulation involved;

- Dry powder mixing
- Wet massing of powder with binder solution and wet granulation
- Drying of wet granules
- Sizing of dried granules

Detailed composition of atenolol granules is given in Table 1 (a similar composition is shown in one of our manuscript [16], where we discussed intrinsic dissolution testing of granules). Microcrystalline cellulose and lactose were used as diluents, primojel as disintegrant and aqueous solution of polyvinyl pyrollidone (PVP k-30) was used as binder for granules preparation. After weighing, all the materials were passed through a screen having 600 μm pore size and loaded to the mixing pan of a laboratory-scale high shear granulator (Yenchen, Taiwan). High shear wet granulator had a three-bladed main impeller (horizontally rotating) and a chopper (vertically rotating). Dry mixing of ingredients was carried at high speed of main impeller (150 rpm) and the chopper at low speed (2200 rpm). Binder solution (aqueous solution of PVP) was taken in hopper and poured slowly, meanwhile, mixing was continued at low speed of impeller (100 rpm) and high speed of chopper (2500 rpm). Binder solution was added slowly at the rate of 1L/20 sec. After addition of binder solution, wet granulation was carried out by rotating both main impeller and chopper at high speed (150 and 2500 rpm respectively). During granulation process, concentration of binder solution and granulation time were selected as process variables and each process variable was studied in different combinations at three levels, as shown in Table 2. Wet granules were dried by hot air stream, using a laboratory scale fluidized bed drier (Yenchen, Taiwan). Before starting drying process, un-heated air was passed through the granules for two minutes, followed by hot air (80 ± 5 °C). After each 3 minutes of drying, filter bags of FBD were shaken for 20 sec, automatically. After completion of drying (25 min), the dried mass was milled by a cone-mill fitted with a 2 mm mesh at 800 rpm. The dried granules were lubricated with magnesium stearate by mixing in a double-cone mixer for 15 min, at 25 rpm.

**Table 1:** Formulation of atenolol granules prepared by high shear wet granulation.

Ingredients	Quantity (%w/w)	Quantity (per tablet)
Atenolol	40.00	100.00
Micro Crystalline Cellulose	30.00	75.00
Lactose*	23.00/18.00/13.00	57.50/45.00/32.50
Primojel	2.00	5.00
Polyvinyl Pyrolidone (PVP k-30)*	5.00/10.00/15.00	12.5/25.00/37.50
Purified Water	Quantity Sufficient	–

*PVP was used in three different concentrations and corrections were made in quantity of lactose, accordingly to keep compression weight constant.

**Table 2:** Different combinations of process variables for preparation of granules by high shear wet granulation.

Trial No	Process Variable-1 (Granulation Time)	Process Variable-2 (Binder Concentration*)
Trial-1	0 (45 sec)	0 (10%)
Trial-2	1 (60 sec)	-1 (5%)
Trial-3	0 (45 sec)	0 (10%)
Trial-4	0 (45 sec)	0 (10%)
Trial-5	1 (60 sec)	1 (15%)
Trial-6	0 (45 sec)	0 (10%)
Trial-7	0 (45 sec)	0 (10%)
Trial-8	-1 (30 sec)	-1 (5%)
Trial-9	-1 (30 sec)	1 (15%)
Trial-10	0 (45 sec)	-1 (5%)
Trial-11	1 (60 sec)	0 (10%)
Trial-12	-1 (30 sec)	0 (10%)
Trial-13	0 (45 sec)	1 (15%)

Data is presented as coded value (actual value), where -1 shows lower, 0 shows mid and +1 shows higher level for both the process variables.

* Binder concentration is given as percentage of the total weight of the granules.

During high shear wet granulation, concentration of binder solution and granulation time were selected as control characteristics of granules, while keeping other processing parameters like speed of main impeller and chopper constant. Speed of impeller and chopper were not tested as a part of this research work as its effect was negligible. Optimum quantity of binder and proper granulation time will result in granules having better characteristics. So, concentration of binder solution and granulation time were taken as process variables for high shear wet granulation and studied in different combinations at three levels (low, mid and high level), as shown in Table 2. Combination of different levels of both the process variables were defined by experimental design, using Design Experts software (version 9.0.5.1; State-Ease Inc, Minneapolis, MN). In the presented manuscript, experimental design was applied for study design only i.e., to get different combinations of process variable for granules preparation, only. It was not to be used for optimization.

Range of granulation time is 30–60 sec and was selected on the basis of process validation (data not shown here). It was sufficient for preparation of granules.

### Characterization of granules on the basis of SeDeM-ODT expert system

SeDeM-ODT expert system is a tool which characterizes pharmaceutical powders and predicts their suitability for tablet preparation [17,18]. Various steps carried out for material evaluation by this system are;

- Determination/calculation of basic parameters
- Conversion of experimental values to “r” values by applying specific factors
- Graphical presentation of results
- Calculation of indices

#### Determination of basic parameters of SeDeM-ODT expert system

According to SeDeM-ODT expert system 15 basic parameters were determined for granules under study [18] according to official/reported methods or calculated on the basis of other basic parameters. Procedures for determination of basic parameters are as under;

- **Bulk Density**: For determination of bulk density, weighed quantity of granules was taken in a graduated cylinder, measured its volume and from the ratio of mass to volume, bulk density was calculated [19].
- **Tapped Density**: Tapped volume of granules was determined by taking weighed quantity of granules and tapping manually until its volume became constant. This final volume was noted as tapped volume. Values of mass of powder blend and tapped volume were used for calculation of tapped density [19].
- **Inter Particle Porosity**: Inter particle porosity was calculated on the basis of the values of bulk and tapped density, using the equation mentioned in Table 3.
- **Car’s Index**: Values of tapped density and bulk density of granules were used for calculation of Car’s index, as per USP [19].
- **Cohesion Index**: Cohesion index is the crushing strength of granules compressed under maximum pressure without capping and lamination [18]. For determination of cohesion index, compacts of granules were prepared under a compression pressure below the level of capping and lamination. Crushing strength of the compacts (n = 10) was measured and their average was taken as cohesion index.
- **Hausner Ratio**: Values of tapped density and bulk density were used for calculation of Hausner ratio, as per USP [19].
- **Angle of Repose**: Funnel method, as prescribed by USP [19] was used for determination of angle of repose, using equation mentioned in Table 3. Experiment was performed in triplicate and mean vales was taken (n = 3).
- **Powder/granules Flow**: For determination of granules flow, granules (100 g) were allowed to flow from a funnel, fitted 3 cm above the table surface and time taken by granules to flow was noted. Average value of three determination was considered (n = 3) [20].
- **Loss on Drying**: For determination of loss on drying, granules (1 g) were taken in to the pan of halogen moisture analyzer (Mettlor Toledo, Switzerland). Heat was applied for 5 min and value of percent weight loss was noted. Experiment was performed in triplicate and mean value was considered (n = 3).
- **Hygroscopicity**: For determination of hygroscopicity, granules were weighed and exposed to high relative humidity 75 ± 5%, in a climatic chamber. Granules were re-weighed after 24 h and value of percent weight gain was taken as hygroscopicity [18].
- **Particle Size Distribution**: Particle size distribution of granules was determined by a mechanical sieve shaker (Endecott, England), equipped with standard meshes (850, 600, 425, 300 and 250 μm pore size) in descending order of pore size. Weighed quantity of granules (100 g) was taken on upper most sieve (850 μm pore size) and sieve shaker was shaken mechanically for specified time (5 minutes) and amount retained over each mesh was determined [18].
- **Homogeneity Index**: Procedure proposed by European pharmacopoeia [20] was used for determination of homogeneity index of granules, by a mechanical sieve shaker (Endecott, England), equipped with standard meshes (850, 600, 425, 300, 250 and 50 μm pore size) in descending order of pore size. Weighed quantity of granules (100 g) was taken on upper most sieve (850 μm pore size) and sieve shaker was shaken mechanically for specified time (5 minutes). Amount retained over each mesh and passing through mesh with 50 μm pore size were determined. Homogeneity index of granules was calculated by the equation given in Table 3.
- **Effervescence Time**: Effervescence time of the compacts was determined according to USP [19]. Compacts of granules were prepared under maximum pressure and a compact was taken in a beaker containing water (200 mL). Noted the time taken by compact to completely disintegration, without any agitation. Effervescence time was determined for six tablets (n = 6) and mean value was considered.
- **Disintegration Time with Disk**: USP tablet disintegration apparatus (Pharma Test, Germany) was used for determination of disintegration time of compacts of granules (compressed under maximum pressure), as per official compendia [19]. Purified water was used as disintegration media, held at 37 ± 2 °C. One compact, along with a disc, was taken in each cylinder of basket and rack assembly. Time taken for disintegration of compact was noted and average value of six compacts (n = 6) was considered as disintegration time.
- **Disintegration Time without Disk**: Disintegration time was determined as described in the previous section, without using disc [19].

**Table 3:** Basic parameters, range of acceptable limits and applied factors of SeDeM-ODT experts system.

Factor/Incidence	Parameter	Symbol	Unit	Equation	Limits	Applied Factor	Ref.
Dimension	Bulk Density	Da	g/mL	Da = M/Va	0–1	10V	[17,18]
	Tapped Density	Dc	g/mL	Dc = M/Vc	0–1	10V	
Compressibility	Inter Particle Porosity	Ie	–	Dc–Da/Dc x Da	0–1.2	10V/1.2	
	Carr’ Index	Ic	%	100 x (Dc–Da)/Dc	0–50	V/5	
	Cohesion Index	Icd	N	Experimental	0–200	V/20	
Flow ability/Powder flow	Hausner Ratio	IH	–	Dc/Da	3–1	(30–10V)/2	
	Angle of Repose	(α)	°	tan ^**-1**^(h/r)	0–50	10 –(V/5)	
	Powder Flow	t"	S	Experimental	0–20	10 –(V/2)	
Lubricity/Stability	Loss on Drying	%HR	%	Experimental	0–10	10 –V	
	Hygroscopicity	%H	%	Experimental	0–20	10 –(V/2)	
Lubricity/Dosage	Particles <50μm	%Pf	%	Experimental	0–50	10 –(V/5)	
	Homogeneity Index	Iθ	–	Fm/100 + ΔFmn	0–2 x 10^−2^	500V	
Disgregability	Effervescence Time	DE	Min	Experimental	0–5	(5 –V) x 2	
	D. Time with Disk	DCD	Min	Experimental	0–3	(3 –V) x 3.33	
	D. Time without Disk	DSD	Min	Experimental	0–3	(3 –V) x 3.33	

Ref.; Reference.

V; Experimental/Calculated Value.

D. Time; Disintegration Time.

Basic parameters of SeDeM-ODT expert system, their symbols, units and acceptable limits are mentioned in Table 3.

#### Conversion of experimental values (V) to radius values (r) and graphical presentation of results

**Fig 1:**
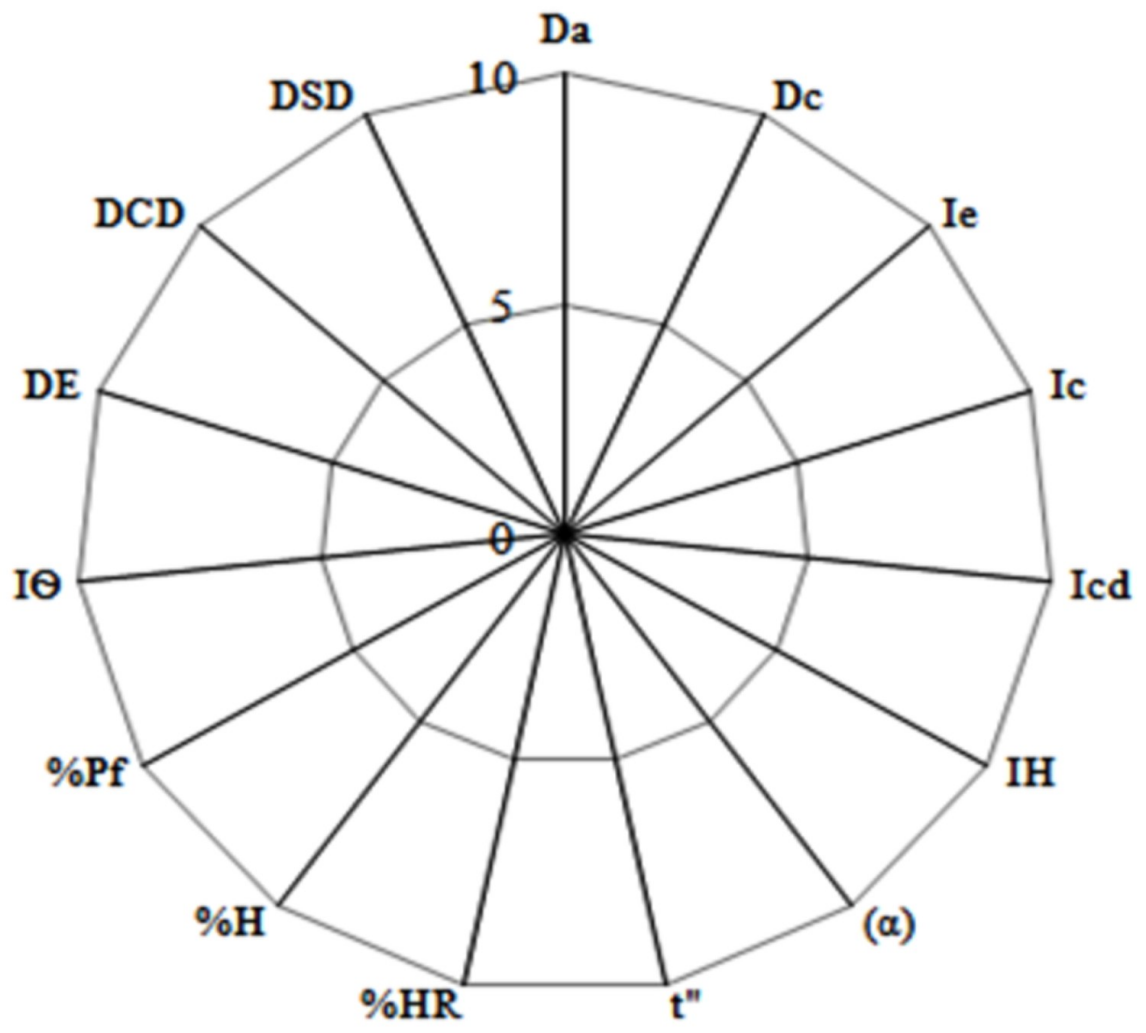
Blank SeDeM-ODT diagram presenting all the basic parameters included in the characterization. Maximum value of “r” is 10, with 5 as lower acceptable limit. Any value above 5 and close to 10 will show better results for the said parameter.

A SeDeM-ODT diagram was constructed, based on the values of basic parameters. Values of the basic parameters were transformed to “r” values by applying factors, which represent radii. Radius value was connected to linear segment to get a SeDeM-ODT diagram. The lowest value of a radius is 0 and 10 is the highest, while 5 is the minimum acceptable value [17]. A representative SeDeM-ODT diagram is shown in Fig 1.

#### Calculation of indices

Optimum mechanical strength, disintegration behavior and rheological characteristics of granules were estimated on the basis of following indices [17,18] calculated using “r” values of determined parameters.

*Parametric index*. Parametric Index is ratio of number of parameters having “r” values equal to or greater than 5 to the total number of parameters determined during the study. Parameter Index was calculated using following equation;

$$I.\;P=\frac{No.\;P\geq 5}{No.\;Pt}$$ (1)

In Eq 1, No. P ≥ 5 is the number of basic parameters having an “r” value higher than or equal to 5 while No. Pt is the total number of parameters included in the study.

*Parameter profile index*. Mean of “r” values of all the basic parameters is called parameter index profile and is calculated by ratio of sum of “r” values of all the parameters and total number of parameters included in the study.


$$IPP=\frac{Sum\;of\;"\text{r"}\;values\;of\;all\;the\;basic\;parameters}{Number\;of\;basic\;parameters\;included\;in\;the\;study}$$ (2)

Its minimum acceptable limit is 5.

*Good compressibility and bucodispersibility index*. Good compressibility and buccodispersibility index (IGCB) is the product of parameter profile index and reliability factor;

I.G.C.B=IPPxf (3)

Where *f* is reliability factor and its value depends upon number of basis parameters included in the study as follows;

For Infinite number of parameters, *f* = 1 (Maximum value)

For 15 parameters, *f* = 0.971

For 12 parameters, *f* = 0.952

For 08 parameters, *f* = 0.900

### Preparation of tablets from granules prepared under different trials

Lubricated granules prepared under different operational parameters were compressed using rotary compression machine (ZP-21, China) fitted with 10.5 mm round flat faced punches, with bisection line on one side. The pitch circle diameters of the rotary compression machine was 292 mm while its speed was 34 rpm. The calculated dwell time was 51 ms. Compression parameters were kept constant for preparation of tablets from all trial. Compression weight of the tablets was 250 mg/tablet and at least 300 tablets were compressed for each trial.

### Characterization of tablets

Granules were compressed to tablets and were characterized for various quality control parameters, as per USP.

#### Physical parameters of tablets

Thickness of randomly selected tablets (n = 10) was measured with a digital hardness and thickness tester (Pharma Test, Germany) and results were presented as mean ± standard deviation. Weight variation test was performed as per official compendia [19] by randomly selecting tablets (n = 20), weighed individually and their mean was calculated. Weight variation was calculated on the basis of the difference between individual and average weights.

#### Drug content of tablets

Drug content of tablets was determined according to USP [19], using mixture of an aqueous phase and methanol (7:3 by volume). For preparation of aqueous phase, sodium phosphate (0.71 g) was dissolved in an aqueous solution of sodium 1-heptanesulfonate (1.1 g in 700 mL of water). Dibutylamine (2 mL) was added, and pH was adjusted to 3 with phosphoric acid (0.8 M). Methanol (300 mL) was mixed with the aqueous phase, filtered through a 0.45 μm filter paper and degassed. Stationary phase consisted of a Hypersil ODS C-8 column (250 mm x 4.6 μm, 5 μm). Tablets (n = 10) were taken in a volumetric flask (1000 mL), mobile phase (500 mL) was added and sonicated till complete disintegration of tablets. Volume was made up to the mark with mobile phase and mixed well. An aliquot was taken from the mixture, centrifuged at 4000 rpm for 5 min and supernatant was diluted with mobile phase to get a concentration of 0.01 mg/mL. Standard solution was prepared by dissolving known quantity of atenolol in mobile phase to get same concentration. Equal volumes of both sample and standard solutions were injected to HPLC and their chromatogram were recorded. Flow rate of mobile phase was 1 mL/min and eluent was detected at 226 nm. Data was analyzed by TotalChrom workstation software (version 6.3.1). Drug content was calculated using following equation;

$$Percent\;Drug\;Content=\frac{Peak\;area\;of\;sample\;solution}{Peak\;area\;of\;standard\;solution}\;x\;100$$ (4)

Drug content was determined in triplicate (n = 3) for each sample and mean value was taken.

#### Wetting time of the tablets

Wetting time of tablets was determined by placing a tablet on filter paper (Whatman Grade 2; Sigma Aldrich) previously soaked in purified water. A tablet was considered completely wet when its upper surface became wet and time taken for complete wetting was taken as its wetting time. Determination of wetting time was made in triplicate for each trial and their mean and standard deviation were calculated (n = 3).

#### Mechanical strength of tablets

USP recommends determination of crushing strength, specific crushing strength, tensile strength and friability test for evaluation of mechanical strength of tablets [19]. Tablets (n = 10) were randomly selected from each formulation and their crushing strength was measured by tablet hardness and thickness tester (PharmaTest, Hamburg, Germany). Average crushing strength of tablets and their standard deviation were calculated.

Mean values of crushing strength and thickness of tablets were used for calculation of tensile strength and specific crushing strength, using the equations proposed by USP [19];

$$Ts=\frac{2F}{\pi DH}$$ (5)

and

$$\tau =\frac{F}{HD}$$ (6)

Where Ts is tensile strength (kg/mm^2^), τ is specific crushing strength (kg/mm^2^), F is crushing strength (kg), D is the diameter (mm) and H is thickness of tablets. While π is constant of proportionality and its value is 3.143.

For determination of friability, tablets (≈ 6.5 g) were randomly taken from each formulation and de-dusted. De-dusted tablets were loaded to the rotating drum of friabilator and rotated at 25 rpm for 4 min [19]. After completion of rotations, tablets were checked for physical defects (breakage, chipping, capping and lamination), re-weighed and weight loss was calculated.

#### Disintegration behavior of tablets

USP tablet disintegration testing apparatus (Pharma Test, Germany) was used for determination of disintegration of tablets, as per official compendia [19], using purified water as a media. Temperature of media was equilibrated at 37 ± 2 °C and one tablet was taken in each cylinder of basket and rack assembly. Disk was used during determination of disintegration time of tablets. Time taken for disintegration of tablet was noted and their average and standard deviation were calculated (n = 6).

#### Determination of dissolution rate

*In vitro* drug release was determined according to USP [19], using 0.1 N acetate buffer (pH 4.6) as dissolution media. Dissolution media (900 mL) was taken in each flask of dissolution apparatus, equilibrated to 37 ± 2 °C and agitated with USP apparatus II (peddle) at 50 rpm. After specified time intervals (0, 5, 15, 30 and 60 min), sample were taken and quantified for atenolol content by HPLC. Details of chromatographic conditions and mobile phase are mentioned in the Section “Determination of drug content”. After sampling, same volume of fresh dissolution media was added.

## Results and discussion

### Characterization of atenolol powder

**Fig 2:**
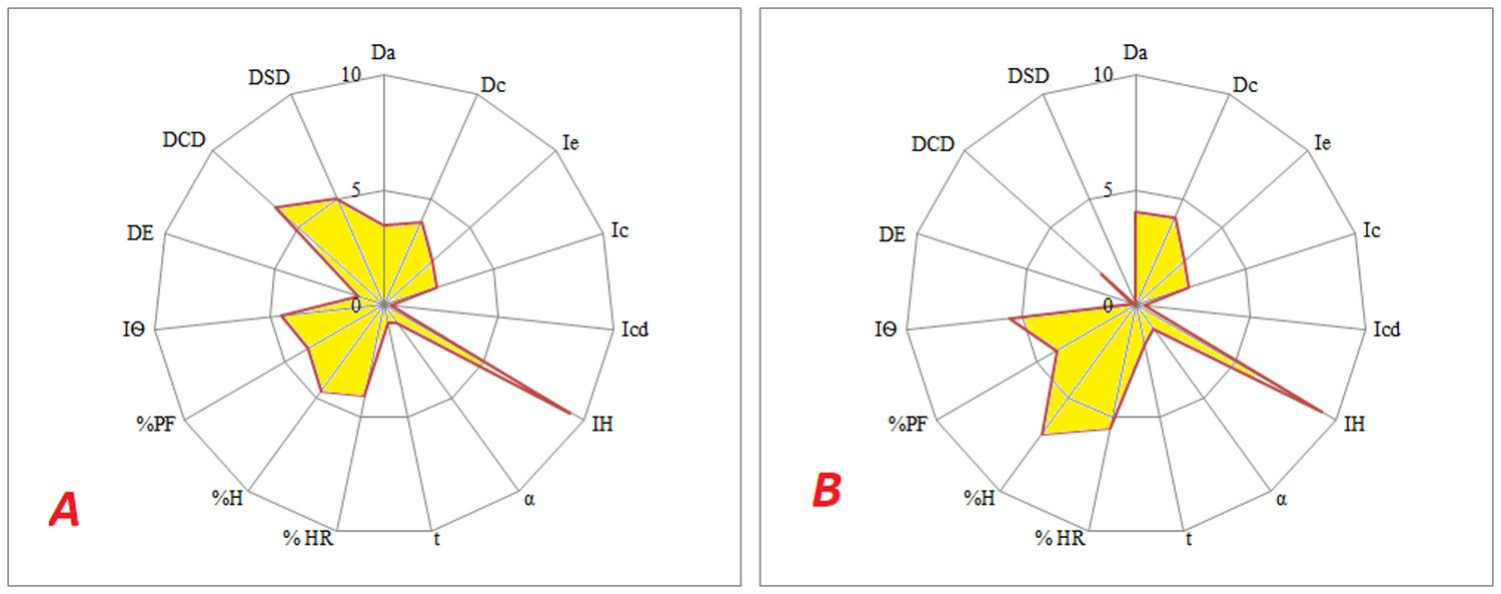
SeDeM-ODT diagrams for atenolol (A) and its blend with other excipients (B).

Atenolol is white to off-white crystalline powder and has good solubility in water [21]. Characterization of atenolol powder on the basis of SeDeM-ODT expert system showed that atenolol was lacking main characteristics (flow and mechanical strength) required for tablet preparation. Flow characteristics of atenolol were estimated on the basis of its angle of repose and flowability which were further supported by Car’s Index and Hausner ratio. The “r” values for angle of repose and powder flowability were below the acceptable limit (˂5), indicating its poor rheological characteristics. Furthermore, poor flow of atenolol powder was confirmed by lower incidence value of the dimension factor (flowability/powder flow), as shown in Table 4. Atenolol was found to be highly hygroscopic and 10.69 ± 0.38% (n = 3) weigh gain was observed upon exposure to high relative humidity (45 ± 5%). Atenolol is stable compound and it does not degrade with high humidity [21] so there was no need to control humidity in processing area. Average hardness of atenolol compact was 0.26 ± 0.19 Kg (n = 10), indicating its poor compressibility. Disintegration behavior of atenolol compact was evaluated on the basis of disgregability factor which was based on effervescence time, disintegration time with disc and disintegration time without disc. Disintegration of atenolol compacts was controlled by its compressibility and water solubility. Generally, material with poor compressibility results in compacts of low mechanical strength, which can easily disintegrate and same was observed with atenolol. All the parameters (disintegration time with disk, disintegration time without disk and effervescence time) showed values within acceptable range because of low mechanical strength of the compacts and water soluble nature of atenolol. SeDeM-ODT diagram of atenolol has low shaded area (Fig 2), with IGCB (4.71) value below acceptable limit (≥5), indicating that most of the factors needed to be improved. Furthermore, some excipients with good wicking properties will be needed to improve its disintegration behavior, which may be adversely affected by increase in mechanical strength.

**Table 4:** Characterization of atenolol powder and its blend with other excipients on the basis of SeDeM-ODT expert system.

Factor/Incidence	Parameter	Atenolol*	Powder Blend*
Dimension	Bulk density (Da)	3.49	4.01
	Tapped density (Dc)	3.96	4.13
Compressibility	Inter particle porosity (Ie)	2.84	2.83
	Car’s Index (Ic)	2.37	2.37
	Cohesion Index (Icd)	0.267	0.346
Flow ability/Powder flow	Hausner ratio (IH)	9.35	9.33
	Angle of repose (α)	0.94	1.27
	Powder flow (’t’)	0.83	1.80
Lubricity/Stability	Loss on drying (%HR)	4.04	5.47
	Hygroscopicity (%H)	4.66	6.96
Lubricity/Dosage	Particles < 50 (%PF)	3.78	3.97
	Homogeneity Index (IѲ)	4.50	5.50
Disgregability	Effervescence Time (DE)	1.21	0.14
	D Time with disk (DCD)	6.33	1.99
	D Time without disk (DSD)	5.06	0

*Data is presented as “r” values, obtained by applying specific factors to experimental/calculated values.

Atenolol was mixed with rest of the ingredients as per Table 1 and powder blend was evaluated for its suitability for compression. Powder blend had relatively better flow and compressibility than pure atenolol powder but still unsuitable for compression as most of the parameters had values below the acceptable limit. Poor compressibility and water soluble nature of ingredients (atenolol, micro crystalline cellulose, lactose and primojel) resulted in better disintegration of the compact and higher value of “disgregability factor”. Smaller shaded area of SeDeM-ODT diagram and lower IGCB value of powder blend showed that most of the parameters still needed to be improved. Dose of atenolol is high (100 mg/tablet) and large quantity of other excipients (like diluents) will be required to mask its poor flow and compressibility. The only solution is granulation of atenolol powder.

### Granulation and characterization of granules

Powder blend containing atenolol, MCC and lactose were granulated by high shear granulator (Yenchen, Taiwan) using aqueous solution of PVP as binder. Objective of the granulation process was to get granules suitable for compression i.e. with optimum flow and compressibility, without compromising their disintegration behavior and drug release so that tablets with better quality attributes can be prepared. Mechanical strength of granules is inversely proportional to their disintegration behavior and drug release [22]. Increase in mechanical strength of granules will decrease its disintegration behavior and vice versa. During wet granulation process, main process variables for controlling granules characteristics are;

- Concentration of binder solution
- Granulation time

**Fig 3:**
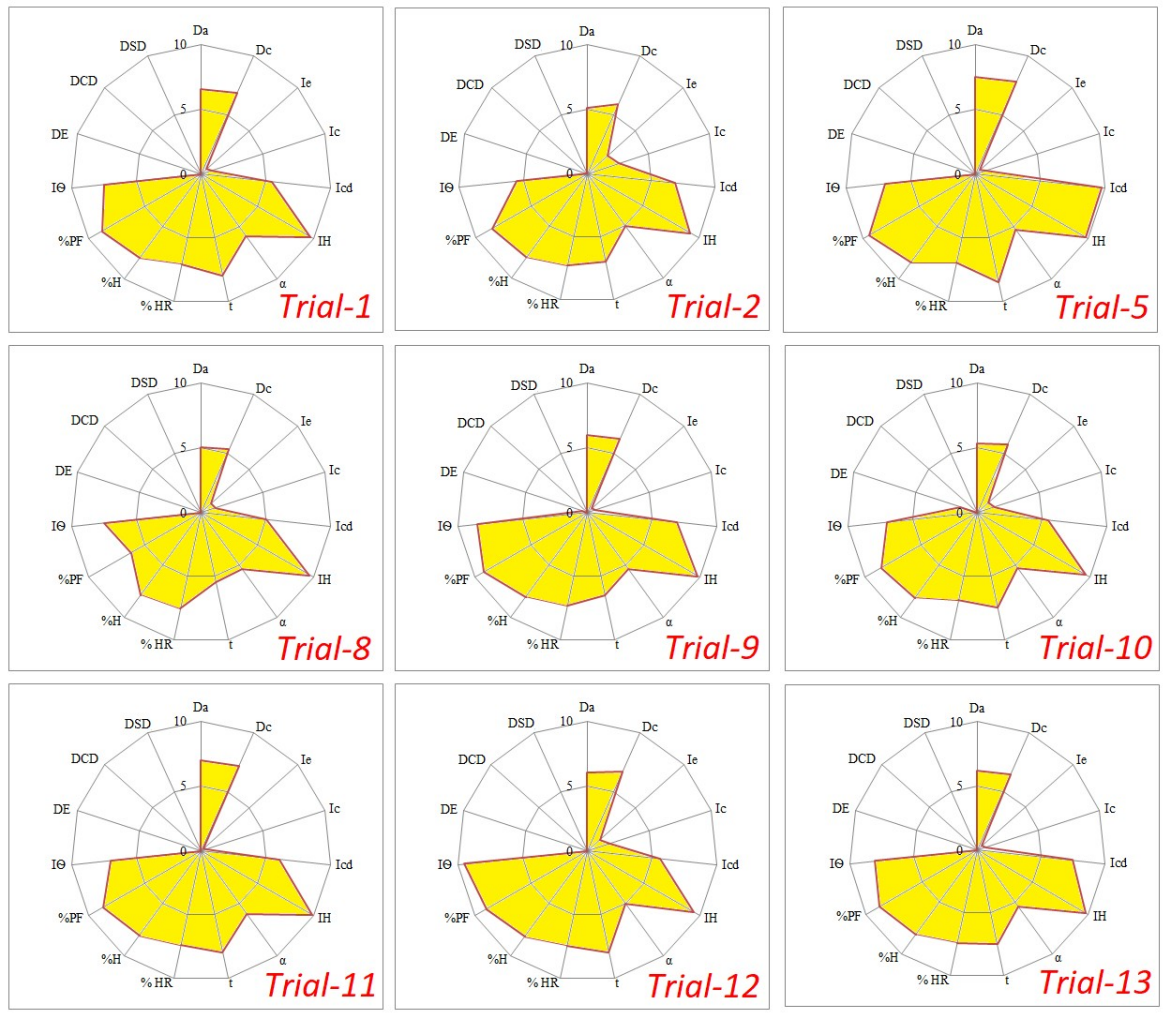
SeDeM-ODT diagrams of granules prepared under different levels of process variables (binder concentration and granulation time) by high shear wet granulation method.

Both the parameters have positive effect on mechanical strength and flow of granules while adversely affect their disintegration behavior and drug release. To get granules with desired characteristics (flow, compressibility and disintegration), both the parameters should be optimized. So granulation was carried out according to the experimental design, presented in Table 2. Granules prepared under each set of process variables and their suitability for compression was evaluated by SeDeM-ODT expert system. Different parameters indicating characteristics of granules were determined/calculated and comparison was made on the basis of different indices like IP, IPP and IGBC. Results showed that granules having optimal characteristics were obtained in Trial-5, which was processed with 15% binder concentration and 60 sec granulation time. Most of the parameters had “r” values above the acceptable limits and SeDeM-ODT diagram had a larger shaded area (Fig 3). Similarly values of IP, IPP and IGCB were also within the acceptable range (5–10), indicating their suitability for compression. During wet granulation by high shear granulator a trend was observed that increase in binder solution and granulation time resulted in granules with higher mechanical strength and better flow. Furthermore, it decreased disintegration behavior and drug release which was in accordance with reported literature. In the present study, aqueous solution of PVP was used as binder in three different concentrations (5, 10 and 15%). It is a polymeric material with strong binding action [23]. At higher concentration of binder solution, powder particles are held strongly, resulting in higher mechanical strength. Higher concentration of binder solution also decreases the proportion of fine powder in granules which has a positive impact on mechanical strength and flow. So all the trials with higher concentration of binder solution exhibited better flow and mechanical strength. IGCB values of granules prepared with higher concentrations of binder solution were higher than those containing lower concentration, as depicted in Table 5.

**Table 5:** The “r” values of granules prepared under different process variables (granulation time and binder concentration) by high shear granulation.

Parameter (symbol)	Trial-1	Trial-2	Trial-5	Trial-8	Trial-9	Trial-10	Trial-11	Trial-12	Trial-13
Bulk density (Da)	6.58	5.13	7.49	5.01	5.98	5.36	7.01	6.08	6.19
Tapped density (Dc)	6.89	5.89	7.83	5.33	6.19	5.78	7.18	6.71	6.43
Inter particle porosity (Ie)	0.567	2.09	0.483	1	0.475	1.133	0.283	1.283	0.5
Car’s Index (Ic)	0.9	2.58	0.868	1.2	0.678	1.454	0.476	1.878	0.746
Cohesion Index (Icd)	5.5165	6.8825	9.75	5.09	6.95	5.46	6.07	5.64	7.435
Hausner ratio (IH)	9.765	9.25	9.775	9.68	9.825	9.61	9.88	9.48	9.8
Angle of repose (α)	5.914	5.062	5.284	5.378	5.42	5.274	5.962	5.038	5.406
Powder flow (’t’)	8	7	8.5	5.5	6.5	7.5	8	8	7.5
Loss on drying (%HR)	7.09	7.31	6.99	7.57	7.33	6.92	7.42	7.46	7.41
Hygroscopicity (%H)	7.985	8.03	8.445	7.855	8.04	8.12	8.07	8.16	8.145
Particles < 50 (%PF)	8.778	8.522	9.408	6.212	9.168	8.562	8.714	8.944	8.804
Homogeneity Index (IѲ)	7.5	5.5	7	7.5	8.5	7	7	9.5	8
Effervescence Time (DE)	-5.66	-7.04	-8.92	-0.78	0.44	1.42	-6.12	-7.46	-8.24
D Time with disk (DCD)	-1.233	-1.699	-7.299	-0.3333	1.167	2.299	-5.333	-6.066	-6.599
D Time without disk (DSD)	-7.366	-9.232	-13.632	-7.233	-2.366	-0.633	-9.766	-10.3656	-13.099

Note; Trial-1 was in quintuplicate and was presented once.

During wet granulation powder coalescence and growth as well as breakage of larger aggregates take place, until a uniform granule size distribution is achieved. In a high shear granulator, wet massing is carried out by main impeller (rotating at speed of 60–800 rpm) and a chopper (rotating at 500–3500 rpm). During rotation of main impeller, collision of particles with impeller, wall of the mixer and with other particles takes place, resulting in granules with smaller size distribution, low porosity and higher density which have good mechanical strength. It has been reported that mean granule size is reduced by higher speed of chopper for longer time [24]. The effect of impeller and chopper on granulation is increased by increasing duration of wet massing i.e. granulation time. Granulation time has both positive and negative effect on granules characteristics *i*.*e*., increase in granulation time;

- Increases rheological characteristics of granules
- Loss of tablet ability

Increase in granulation time usually increases the effect of main impeller and chopper resulting in reduced granular size, high density and higher mechanical strength. So Trial-5 (processed at higher level of both the process parameters) exhibited better flow and mechanical strength, while its disintegration was poor (as depicted by the value of its disgregabiliy factor presented in Table 6).

**Table 6:** Mean incidence values calculated for incidence factors on the basis of SeDeM-ODT expert system.

Trial No.	Dimension	Compressibility	Flowability/Powder Flow	Lubricity/Stability	Lubricity/Dosage	Disgregability
Atenolol Powder	3.725	1.825	3.707	4.348	4.141	4.2
Powder Blend	4.07	1.85	4.134	6.215	4.734	0.713
Trial-1	6.735	2.33	7.893	7.538	8.139	0
Trial-2	5.51	3.85	7.104	7.67	7.011	0
Trial-5	7.66	3.7	7.853	7.718	8.204	0
Trial-8	5.17	2.43	6.853	7.712	6.856	0
Trial-9	6.085	2.701	7.25	7.685	8.834	0.147
Trial-10	5.57	2.68	7.46	7.52	7.781	0.473
Trial-11	7.095	2.276	7.947	7.745	7.857	0
Trial-12	6.395	2.934	7.506	7.81	9.222	0
Trial-13	6.31	2.894	7.569	7.778	8.402	0

SeDeM-ODT diagrams for granules prepared under different trials are presented in Fig 3.

IGCB values of most of the trials were found below the acceptable limits (<5) because of lower values of disgregability factor. Granulation increased mechanical strength of granules leading to poor disintegration and lower values of disgregability factor resulted in lower values of IGCB. There are two versions of the used expert system;

- SeDeM Expert System
- SeDeM-ODT Expert System

**Fig 4:**
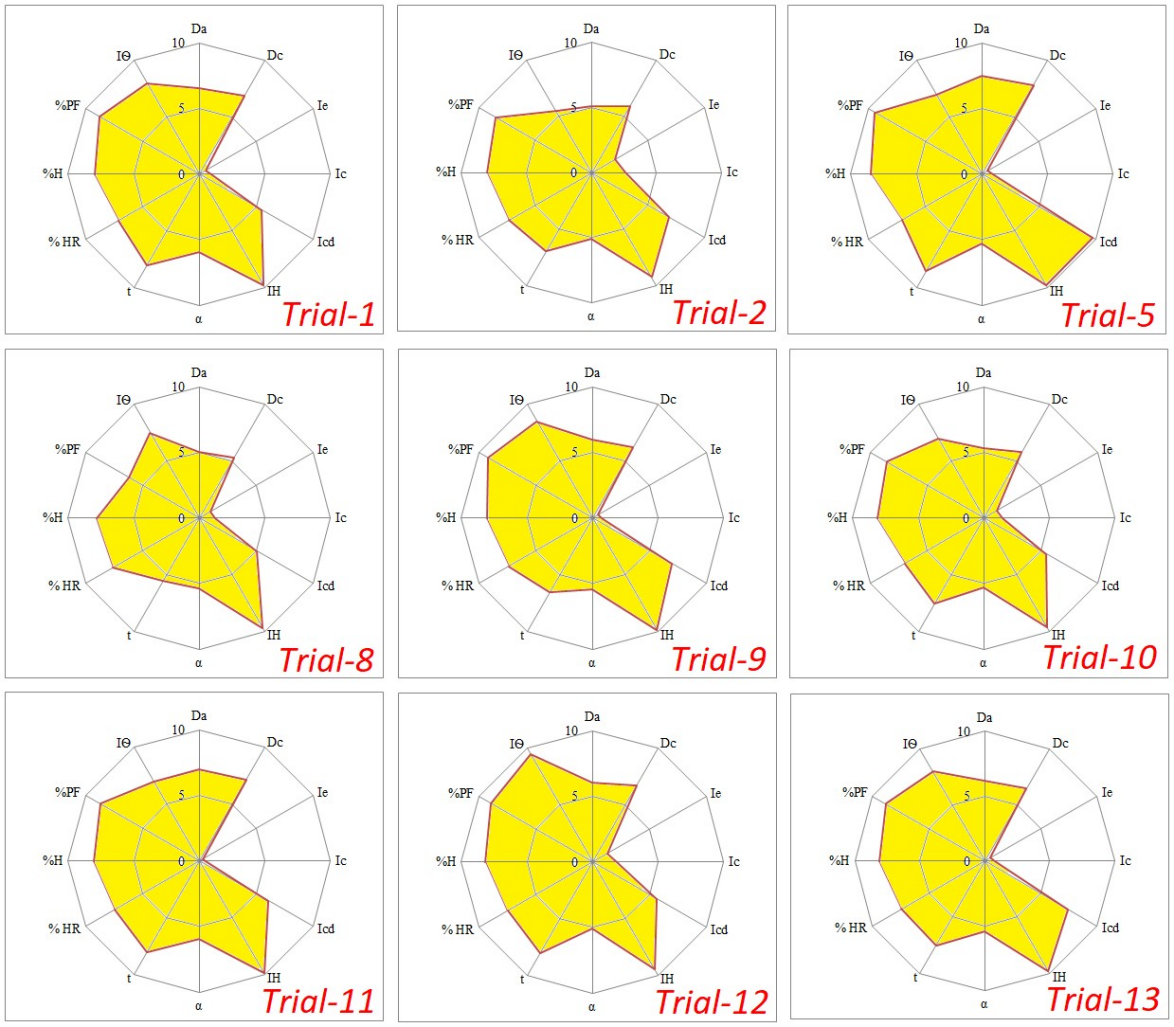
SeDeM diagrams of granules prepared under different levels of process variables. SeDeM expert system predicts suitability of material for compression only.

SeDeM expert system is used for determination of suitability of material for compression and is based on parameters related to flow and compressibility. SeDeM-ODT expert system is based on parameters related flow, compressibility and dispersibility. Along with flow and compressibility, it also contains parameters related to disintegration behavior of the material like its effervescence time, disintegration time with disk and disintegration time with disk. It has been mainly applied for determination of buccodispersibility of ODTs. Here both the systems has been used for prediction of quality attributes of tablets because high shear granulation increases both flow and compressibility and has a negative effect on disintegration behavior of tablets. So SeDeM-ODT expert system was used to predict disintegration behavior of tablets, along with their mechanical strength and physical parameters. Indices were calculated on the basis of both versions of expert system (SeDeM and SeDeM-ODT) and results are shown in Table 7. Evaluation of granules on the basis of SeDeM expert system (rather than SeDeM-ODT) showed that granules from all the trials were suitable for compression. Values of Index of Good Compressibility (IGC) were within the acceptable range and their SeDeM diagrams had larger shaded area, as shown in Fig 4. So it can be predicted from results that tablets prepared from granules (processed under different levels of process parameters) will have sufficient mechanical strength and their disintegration time will not meet the official requirements. High quantity of disintegrants will be needed to achieve acceptable disintegration of tablets.

**Table 7:** Values of different indices calculated on the basis of SeDeM and SeDeM-ODT experts system.

Trial No	SeDeM-ODT Expert System			SeDeM Expert System		
	IP	IPP	IGCB	IP	IPP	IGC
Atenolol	0.2	3.58	3.47	0.083	3.419	3.255
Powder Blend	0.267	3.34	3.24	0.333	3.999	3.807
Trial-1	0.667	5.03	4.89	0.833	6.29	5.988
Trial-2	0.667	4.88	4.74	0.833	6.104	5.811
Trial-3	0.667	5.03	4.89	0.833	6.29	5.989
Trial-4	0.667	5.03	4.89	0.833	6.29	5.989
Trial-5	0.667	5.45	5.29	0.833	6.819	6.491
Trial-6	0.667	5.03	4.89	0.833	6.29	5.989
Trial-7	0.667	5.03	4.89	0.833	6.29	5.989
Trial-8	0.667	4.49	4.36	0.833	5.61	5.341
Trial-9	0.667	5.03	4.89	0.833	6.26	5.954
Trial-10	0.667	4.91	4.76	0.833	6.014	5.726
Trial-11	0.667	5.07	4.92	0.833	6.339	6.034
Trial-12	0.667	5.21	5.06	0.833	6.514	6.202
Trial-13	0.667	5.09	4.94	0.833	6.364	6.058

IP; Parameter Index.

IPP; Parameter Index Profile.

IGCB; Index of Good Compressibility and Bucco-dispersibility.

IGC; Index of Good Compressibility.

### Evaluation of atenolol tablets

#### Physical characteristics

Theoretical weight of atenolol tablets was 250 mg/tablet. Weight variation was in a narrow range, which was due to better and uniform flow of granules during compression. Thickness of the tablets, prepared under different trials, showed variation on the basis of granules density. Tablets prepared with Trial-5 (highest level of both the process variables) had highest density and resulted in tablets with lowest thickness. Similarly granules prepared under Trial-8 resulted in tablets with highest thickness, because of their low density.

Tablets prepared under different process variables had different wetting time. Wetting time is an indicator of water penetration into the tablet core and increases with increase in mechanical strength of granules. By increasing mechanical strength of granules, their inter particle porosity is decreased, resulting in decreased water penetration to the tablet core. Tablet prepared with granules having higher cohesion index showed longer wetting time and vice versa. Drug contents of all the formulations were within the 98–101% of the claimed quantity (Table 8), which was within the official limits [19].

**Table 8:** Post compression evaluation of atenolol tablets prepared by high shear granulation under different process variables.

Parameter (Unit)	Trial-1	Trial-2	Trial-5	Trial-8	Trial-9	Trial-10	Trial-11	Trial-12	Trial-13
Average Weight (mg)	258.21	252.68	252.91	254.92	256.17	255.36	252.19	254.8	251.98
Weight Variation (%)	±3.5	±2.91	±3.11	±3.12	±3.73	±3.19	±2.85	±3.26	±2.71
Thickness (mm)^a^	3.48 ± 0.31	3.65 ± 0.46	3.34 ± 0.29	3.61 ± 0.52	3.47 ± 0.18	3.68 ± 0.37	3.41 ± 0.39	3.59 ± 0.24	3.43 ± 0.26
Crushing Strength (kg)^a^	9.86	7.19	12.07	5.3	9.12	6.73	10.48	8.63	11.8
Specific Crushing Strength (kg/mm^2^)^b^	0.27	0.188	0.344	0.14	0.25	0.174	0.293	0.229	0.328
Tensile Strength (kg/mm^2^)^b^	0.172	0.119	0.219	0.089	0.159	0.111	0.186	0.146	0.208
Friability (%)	0.45	0.72	0.31	1.3	0.4	0.78	0.39	0.45	0.19
Wetting Time (sec)	195	168	249	139	182	154	201	174	235
Drug Content (%)^c^	98.63 ± 0.78	99.7 ± 0.91	99.17 ± 0.46	99.67 ± 0.38	98.02 ± 0.63	97.68 ± 0.93	99.11 ± 0.52	100.73 ± 0.79	99.82 ± 0.32
Disintegration Time (minute)^d^	8.5 ± 0.91	10.8 ± 0.37	12.65 ± 0.98	6.8 ± 0.29	8.2 ± 1.17	7.5 ± 0.98	10.3 ± 0.34	7.8 ± 0.69	10.9 ± 1.08
Q_15 min_	62.39 ± 0.74	65.19 ± 0.92	21.37 ± 1.19	79.52 ± 1.43	63.18 ± 0.91	68.4 ± 0.73	52.07 ± 1.16	70.02 ± 0.39	48.73 ± 0.83

Q_15 min_; Amount of drug released after 15 min.

^a^; Data is presented as mean ± SD (n = 10).

^b^; Calculations are based on mean hardness and thickness of tablets.

^c^; Data is presented as mean ± SD (n = 3).

^d^; Data is presented as mean ± SD (n = 6).

#### Mechanical strength

Crushing strength of atenolol tablets depended on mechanical strength (cohesion index) of granules. Trials processed under highest level of both the process variables (binder concentration and granulation time) showed highest level of mechanical strength and same behavior was shown by the tablets, prepared from respective granules. The lowest crushing strength was observed for Trial-8, prepared from granules prepared under lowest level of both the process variables. While highest mechanical strength was observed with Trial-5, having higher crushing strength (12.07 kg), tensile strength (0.219 kg/mm^2^) and specific crushing strength (0.344 kg/mm^2^), as shown in Table 8. Friability of the tablets from all the trials was within the permissible range (<1%) and edging or capping was not observed with any trial, indicating their better mechanical strength. In comparison with granulation time, the effect of binder concentration was high on mechanical strength of tablets which may be due to complete coverage of powder particles with binder material and its more binding force.

#### Disintegration time

**Fig 5:**
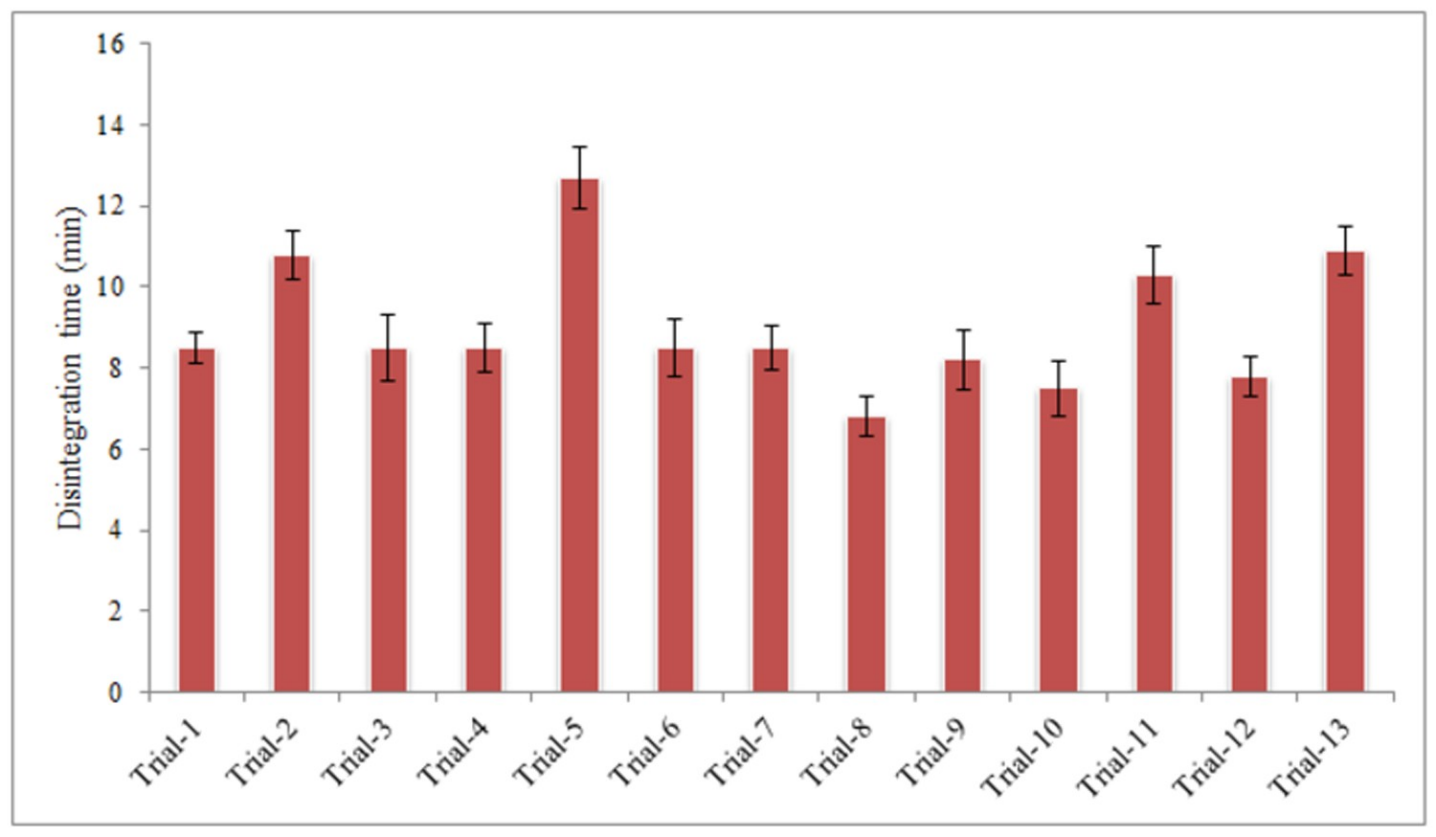
Disintegration time of tablets prepared from granules processed under different experimental conditions.

Disintegration time of tablets from all the trials was within the official limits. For the purpose of comparison disintegration time was determined individually and their mean and standard deviation were calculated (Fig 5). Disintegration time increased with increase in mechanical strength of the granules. Higher disintegration time was observed with tablets prepared from the granules processed at higher level of both the process variables (binder concentration and granulation time) due to poor water penetration into tablet core and relatively larger quantity of disintegrants will be needed for their rapid disintegration.

#### Dissolution rate

**Fig 6:**
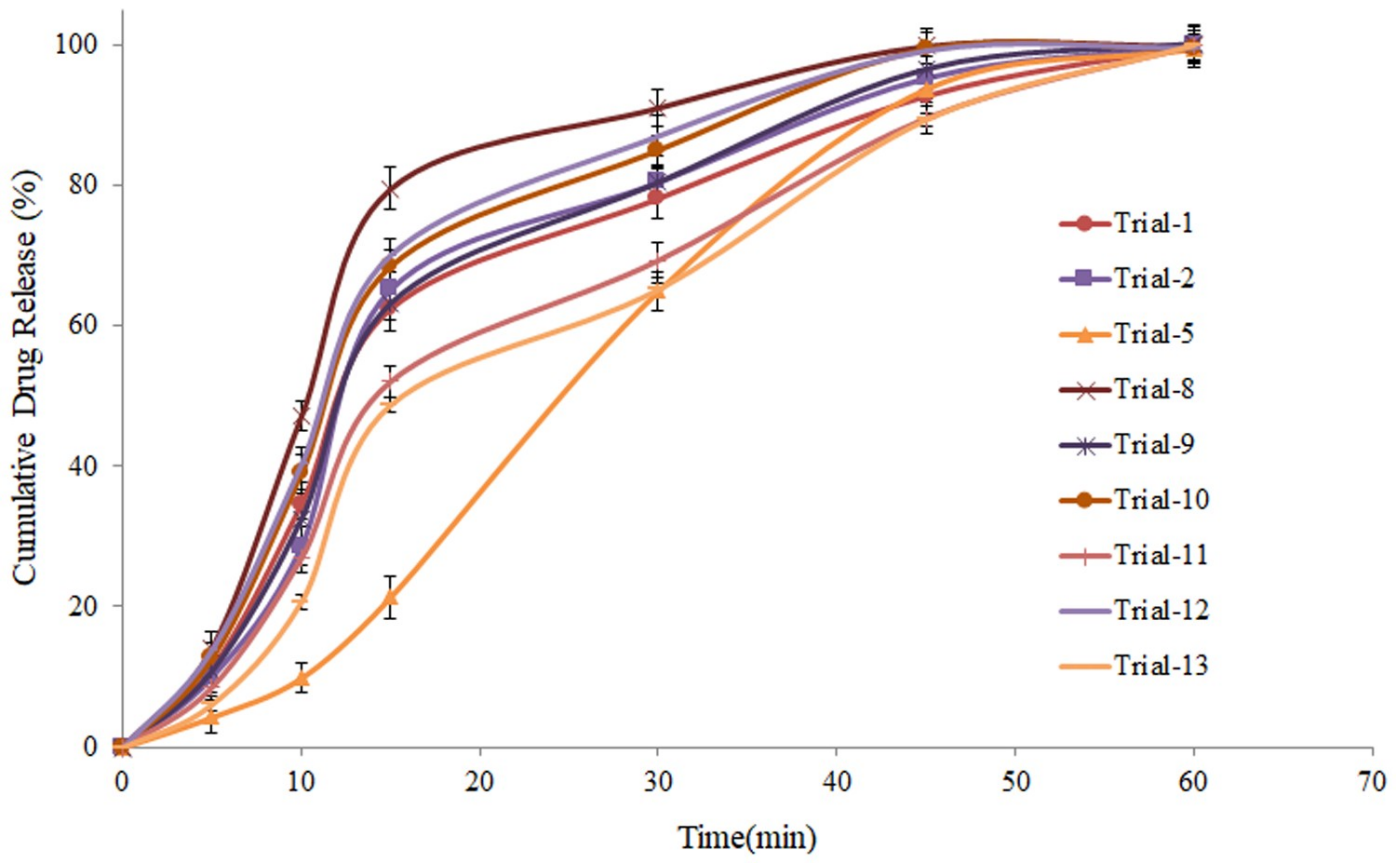
Dissolution rate of atenolol from tablets prepared from granules, processed under different processing parameters by high shear wet granulation.

Dissolution rate was studied according to USP, using 900 mL of acetate buffer (pH 4.6) as dissolution media. Dug release from tablets was negatively affected by both the process variables (binder concentration and granulation time). Highest drug release was observed with Trial-8, processed at lower level of both the process variables, where 79.52 ± 1.29% (n = 3) drug released within initial 15 min. Tablets prepared from granules processed under highest level of both the process variables (Trial-5) showed lowest drug release (21.37 ± 0.98%; n = 3) within 15 min. Increase in binder concentration and granulation time during high shear granulation resulted in higher mechanical strength of granules due to strong bonding of the particles, which needed much time for penetration of dissolution media and subsequent drug release. Moreover, PVP is a polymeric material which forms strong bonds among particles of drug and excipients, delays the dug release. It is evident from the data presented in Fig 6, that tablets prepared from granules with higher cohesion index showed lower drug release and vice versa.

## Conclusion

Aim of the study was to predict quality attributes of tablets prepared by high shear wet granulation technique, on the basis of characteristics of granules. In the present study, granules containing atenolol were prepared under different process variables (concentration of binder solution and granulation time) of high shear wet granulation and properties of the resultant tablets were predicted on the basis of SeDeM-ODT expert system. Granules were compressed and tablets were evaluated for different quality control parameters. Tablets exhibited quality attributes as predicted on the basis of granules characteristics. SeDeM-ODT expert system predicted that both the process variables will increase mechanical strength of tablets and same characteristics were shown by the tablets. It is concluded from study that characterization of granules on the basis of SeDeM-ODT expert system can predict characteristics of tablets.
